# Pulmonary-Esophageal Variceal Bleeding: A Unique Presentation of Partial Cor Triatriatum Sinistrum

**DOI:** 10.1155/2013/538259

**Published:** 2013-12-03

**Authors:** Fortune O. Alabi, Manuel Hernandez, Francis G. Christian, Fred Umeh, Maximo Lama

**Affiliations:** ^1^Department of Critical Care Medicine, Florida Hospital Celebration Health, Celebration, FL 34747, USA; ^2^Department of Radiology, Florida Hospital Orlando, Orlando, FL 32803, USA

## Abstract

Cor triatriatum sinistrum is a rare congenital disorder defined as a division of the left atrium by a diaphragmatic membrane resulting in two left atrial chambers. The membranous division of the atrium can be partial or complete and can affect either atrium, with involvement of the right atrium referred to as cor triatriatum dexter. The presence of fenestrations within the membrane allows for communication and forward passage of blood into the true atrium. Absence of fenestrations leads to early symptomatic engorgement of the lungs. We report the case of a young adult male presenting with recurrent hematemesis due to variceal bleeding. On CT imaging the patient was found to have cor triatriatum sinistrum, with a vertical membrane resulting in total obstruction of the pulmonary venous drainage on the right, with normal pulmonary venous drainage on the left. There was extensive pulmonary-systemic arterial collateralization to the right lung suggesting retrograde filling of the right pulmonary artery with effective flow reversal in the right lung.

## 1. Introduction

Cor triatriatum was first described in 1868 [1]. Since then, reports have described the condition in the pediatric population where presentation is acute and symptomatic. Affected children present with features of congestive heart failure such as decreased cardiac output and pulmonary venous hypertension. With the advent of TTE and TEE, there have been confirmed diagnoses of cor triatriatum presenting in adults with fatigue, dyspnea on exertion, and recurrent infection [2]. When presenting in adulthood, cor triatriatum is mostly isolated; however, the condition has been reported in association with bicuspid aortic valve and atrial septal defects [3].

The unique defect in partial cor triatriatum involving only the right pulmonary veins (with normal flow of left pulmonary veins into left atrium) results in pulmonary venous hypertension and congestion in only the right lung. Subsequent vascular changes of increased mediastinal venous drainage result in development of venous varices. Complete venous obstruction can also result in extensive pulmonary-systemic arterial collateralization providing an effective systemic to pulmonary arterial shunt. In this setting, forward flow to the lung is provided by hypertrophied systemic arteries including phrenic, pleural, aortic, and bronchial artery collaterals, while venous drainage of the lung is successfully provided by retrograde flow within the affected pulmonary artery. To date, there has been no case describing variceal bleeding as a presentation of partial cor triatriatum sinistrum.

## 2. Case Report

**Figure 1:**
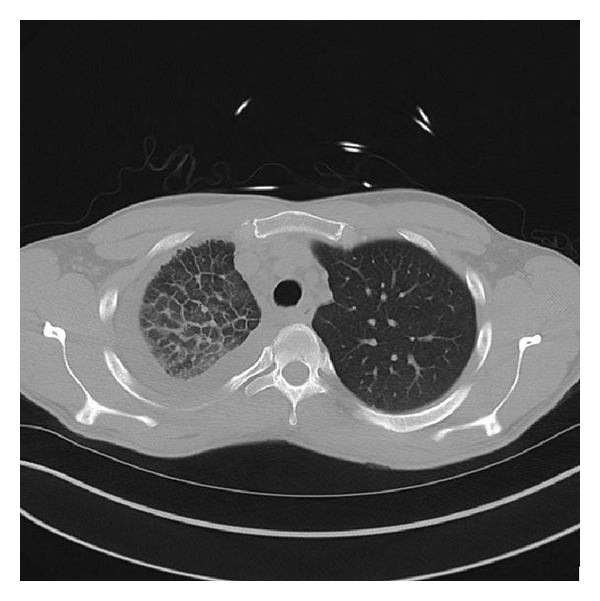
Axial plane showing right lung volume loss, secondary lobules, and thickened septa.

**Figure 2:**
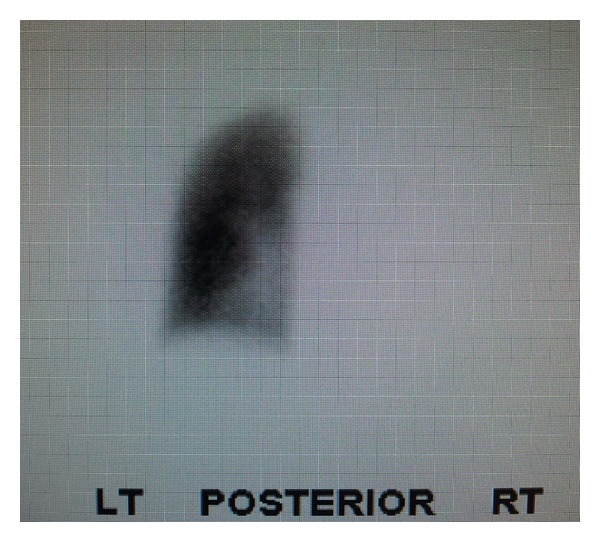
Albumin perfusion scan revealing absence of right lung perfusion.

**Figure 3:**
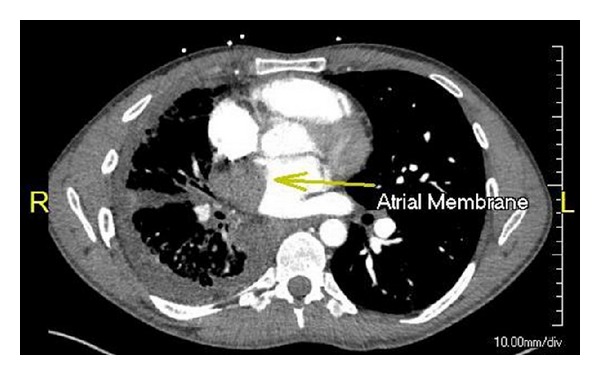
Axial plane showing vertical membrane (yellow arrow) in left atria obstructing right pulmonary venous flow.

**Figure 4:**
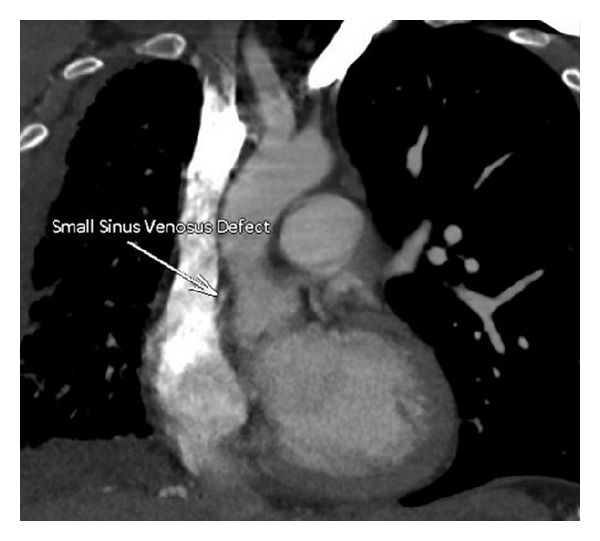
Coronal plane showing sinus venosus collateralization between the hypertrophied systemic vessels and the diminutive right pulmonary artery.

**Figure 5:**
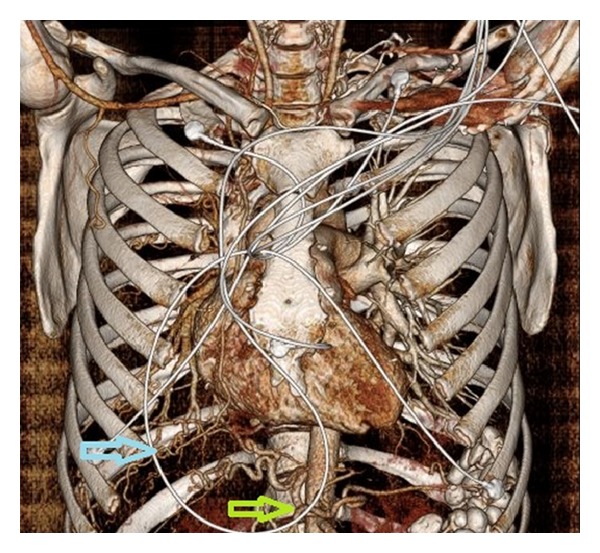
Three-dimensional reconstructed image showing origin of anomalous artery (green arrow) supplying diaphragmatic collaterals. Phrenic-pulmonary arcades and marginal pulmonary systemic shunts (blue arrow) are seen draining via the small right pulmonary artery.

A 26 y/o male was admitted to the critical care service from the emergency department for severe anemia with variceal bleeding. The patient presented with active hematemesis citing a weeklong history of melanotic stool, hematemesis, and lightheadedness. There were no significant past medical or family histories of GI or pulmonary diseases. The patient's social history describes 2 glasses of wine per week for the last 4 years. Physical examination on consultation revealed an alert, young male with mild epigastric tenderness without pulmonary or cardiovascular findings. CT of the abdomen showed supradiaphragmatic “downhill” paraesophageal varices without cirrhosis or splenomegaly and pleural thickening with pleural calcifications. Upper endoscopy revealed numerous 4+ varices from the upper esophagus to the gastroesophageal junction which were banded. High resolution noncontrast CT the following day revealed right lung findings significant for volume loss, septal thickening of the secondary pulmonary lobules, and a “cobble stoning” indicative of pathologic interstitial edema (Figure 1). V/Q scan revealed nearly absent right lung perfusion (Figure 2). Subsequently, a CT of the chest to evaluate for pulmonary vein atresia showed normal left pulmonary veins with near complete functional arterial and venous systemic isolation of the right lung due to chronic right pulmonary vein obstruction. The left atrium was significant for a vertical septation resulting in complete isolation of the right upper and lower pulmonary veins (Figure 3). A small caliber left to right to shunt was noted between the upper portion of the left atrium and the SVC via a patent sinus venosus (Figure 4). Right lung findings were consistent with chronic pleural congestion, extensive arterial collateralization, and pericardial and peridiaphragmatic lymph node enlargement secondary to vascular congestion. The right lung was supplied by numerous hypertrophied intercostal and peripleural collaterals along the periphery in addition to hypertrophied bronchial artery collaterals. Systemic-pulmonary artery collaterals were also identified as arising directly from the lower thoracic and upper abdominal aorta just superior to the celiac trunk. These collaterals provided retrograde filling of the right pulmonary artery via hypertrophied phrenic artery collaterals forming a series of pulmonary-phrenic arcades and shunts along the diaphragm (Figure 5).

The patient was stabilized and transferred to an outside institution where he underwent surgical resection of the obstructing atrial membrane. After surgery, the patients' hemoptysis, hematemesis, and melena resolved. Repeat upper endoscopy demonstrated no significant residual varices and CT bolus tracking images and echocardiography showed normal anterograde flow in right pulmonary artery.

## 3. Discussion

Cor triatriatum sinistrum involves a membranous appendage dividing the left atrium into a proximal (accessory) chamber and distal (true) left atrium. Pulmonary veins returning to the left atria enter the blind ending accessory chamber rather than true left atrium. Without a large orifice of >3 mm or fenestrations in the membrane [4], there will be significantly decreased right lung venous outflow resulting in chronic pulmonary congestion. Subtotal variations of sinistrum may occur as in the presented case, whereby the causative septation affects pulmonary venous drainage from only one lung.

Classification of cor triatriatum into 3 groups was first made by Loeffler [5]. Group 1 has no orifices, in the sinistrum membrane, group 2 has 1 or more small orifices and group 3 has a wide orifice. Groups 1 and 2 present in infants with severe symptomatology and mortality, while group 3, Loeffler postulated, may reach adulthood. This patient likely had no fenestrations in the occluding membrane, having survived till adulthood without significant symptomology due to unilateral involvement and effective left to right shunting. Flow reversal on the right allowed for pulmonary venous return and thus normal lung development.

Unilateral right sided pulmonary vein atresia is the most likely considered differential diagnosis for the case presented. Both conditions have the same physiological incapability of proper forward blood flow from right lung to left atrium. There is physiologic reversal of normal blood flow from the bronchial veins though systemic anastomoses. Normal bronchial venous return occurs through two pathways. First, bronchial veins drain into esophageal veins which flow into the azygous vein and superior vena cava providing return to the right heart. A second pathway is through small bronchopulmonary anastomoses into the pulmonary veins providing deoxygenated return to the left heart, the basis for physiological shunting [6]. Physiologic reversal of flow to the pulmonary veins and increased flow through the esophageal veins results in the formation of “downhill” paraesophageal varices rather than the uphill esophageal varices commonly encountered in portal venous hypertension. Additionally, systemic-pulmonary artery collateralization as seen in this case has also been reported in cases of isolated pulmonary vein atresia [7].

Physiologic similarities between this case of partial cor triatriatum and unilateral right pulmonary vein atresia are manifested in the resemblance of imaging studies. Chest CT demonstrated a small right hemithorax, ground glass pulmonary infiltrates, interlobular septal thickening, and a small caliber right pulmonary artery. V/Q scan revealed nearly absent pulmonary perfusion to right lung with normal ventilation. These findings are almost identical to those reported in previous adult cases of unilateral pulmonary vein atresia [8]. The septated left atrium on CT venous chest and nonatretic pulmonary veins are the only significant differences between imaging studies here and previously reported cases of pulmonary vein atresia.

## 4. Conclusion 

Pulmonary vein atresia is an uncommon but known cause of esophageal varices [9]. When investigating clinical presentations of dyspnea, recurrent infections, and hematemesis, along with concurrent radiological findings significant for pulmonary vein atresia, it would be prudent to consider partial cor triatriatum as a possible differential diagnosis.

## References

[1] Church WS (1868). Congenital malformation of heart: abnormal septum in left auricle. *Transactions of the Pathological Society of London*.

[2] Işılak Z, Cay S, Kardeşoğlu E, Uzun M (2012). Fenestrated cor triatriatum sinistrum: a case report. *Türk Kardiyoloji Derneği Arşivi*.

[3] Hamdan R, Mirochnik N, Celermajer D, Nassar P, Iserin L (2010). Cor Triatriatum Sinister diagnosed in adult life with three dimensional transesophageal echocardiography. *BMC Cardiovascular Disorders*.

[4] Karamlou TB, Welke KF, Ungerleider RM, Brunicardi FC, Andersen DK, Billiar TR (2010). Congenital heart disease. *Schwartz's Principles of Surgery*.

[5] Loeffler E (1949). Unusual malformation of the left atrium; pulmonary sinus. *Archives of Pathology*.

[6] Rhoades RA, Rhoades R, Bell DR (2013). *Medical Physiology, Principles for Clinical Medicine*.

[7] Cao M, Cai H, Ding J, Zhuang Y, Wang Z (2013). Bronchial varices in congenital unilateral pulmonary vein atresia. *American Journal of Respiratory and Critical Care Medicine*.

[8] Heyneman LE, Nolan RL, Harrison JK, McAdams HP (2001). Congenital unilateral pulmonary vein atresia: radiologic findings in three adult patients. *American Journal of Roentgenology*.

[9] Harrison JK, Hearne SE, Baker WA (1996). Esophageal varices in association with unilateral pulmonary vein atresia. *Catheterization and Cardiovascular Interventions*.

